# Development of EBV Related Diffuse Large B-cell Lymphoma in Deficiency of Adenosine Deaminase 2 with Uncontrolled EBV Infection

**DOI:** 10.1007/s10875-024-01712-x

**Published:** 2024-05-17

**Authors:** Logan S. Gardner, Lachlin Vaughan, Danielle T. Avery, Isabelle Meyts, Cindy S. Ma, Stuart G. Tangye, Winny Varikatt, Ming-Wei Lin

**Affiliations:** 1 School of Public Health and Preventive Medicine, Monash University, Clayton, Australia; 2 ICPMR, NSW Health Pathology, Westmead Hospital, Sydney, Australia; 3 The Lowy Cancer Research Centre, Kensington, Australia; 4 Faculty of Medicine, University of Sydney, Camperdown, Australia; 5 Garvan Institute of Medical Research, Darlinghurst, Australia; 6 Department of Immunology and Microbiology, Laboratory for Inborn Errors of Immunity, Department of Pediatrics, University Hospitals Leuven and KU Leuven, Leuven, EU Belgium; 7 Clinical Immunogenomics Research Consortium of Australasia (CIRCA), Darlinghurst, Australia; 8 Faculty of Medicine and Health, St Vincent’s Clinical School, UNSW Sydney, Sydney, Australia; 9 Department of Immunology, Westmead Hospital, Westmead, Sydney, Australia

**Keywords:** Deficiency of Adenosine Deaminase 2, EBV, Diffuse Large B-cell Lymphoma

## Abstract

Deficiency of Adenosine Deaminase 2 (DADA2) patients presenting with primary immunodeficiency are at risk of uncontrolled EBV infection and secondary malignancies including EBV-related lymphoproliferative disorders (LPD). This paper describes the first case of EBV related diffuse large B-cell lymphoma in a patient with DADA2 and uncontrolled EBV infection. Consideration should be given to monitoring for EBV viraemia and to preventative EBV specific therapy in DADA2 and patients with at risk primary immunodeficiencies. A type I interferon (IFN) gene signature is associated with DADA2 though its association with immune dysregulation is unclear.

**Supplementary Information:**

The online version contains supplementary material available at 10.1007/s10875-024-01712-x.

## Introduction

Deficiency of Adenosine Deaminase 2 (DADA2) is a heterogenous disorder due to biallelic mutations in *ADA2* that typically presents in childhood with a vasculopathy similar to polyarteritis nodosa [1]. Typical symptoms are due to consequences of end organ ischaemia, including early onset stroke, bowel ischaemia and constitutional symptoms.

Other prominent clinical phenotypes include autoimmunity, bone marrow failure and immunodeficiency [2]. The presentation of immune deficiency is similar to common variable immune deficiency (CVID) with low serum immunoglobulins (Ig) and poor Ab response to vaccination. However, this may not be clinically apparent until adulthood [2, 3]. Indeed, the presence of associated cellular immune deficiency may not be recognised even though increased susceptibility of ADA2-deficient individuals to viral infections including Epstein Barr Virus (EBV) has been reported [1, 2].

EBV affects up to 90% of the adult population, remaining latent within B cells and intermittently reactivating to infect new B cells [4]. Persistent antigenic stimulation and the production of viral proteins that promote cell survival and growth predisposes affected individuals to the development of EBV-related LPDs including the rare variant lymphomatoid granulomatosis (LG) [4, 5]. This process is accelerated in the setting of impaired cellular immunity predisposing to prolonged EBV viraemia [4, 5]. Additionally chronic EBV viraemia has been demonstrated to be a trigger for immune dysregulation in genetically predisposed hosts [6].

According to the 5th edition of WHO, lymphomas arising in Immunodeficiency and/or immune dysregulation are classified under several subcategories including small B-cell lymphoma, diffuse large B-cell lymphoma (DLBCL), Burkitt lymphoma and classic Hodgkin lymphoma. EBV related DLBCL can have a lymphomatoid granulomatosis like appearance, however according to the current definition, a history of immunodeficiency/dysregulation other than immune senescence excludes the diagnosis of LG [7]. To our knowledge we present the first documented case of Immunodeficiency related EBV positive DLBCL in a patient with DADA2.

## Case Report

A 38-year-old woman initially presented with recurrent sinopulmonary infections, low serum Ig, low class switched memory B cells and an absent response to pneumococcal vaccination. An initial diagnosis of CVID was made and she was commenced on Ig replacement. Her clinical history was significant for depression and Thalassaemia by electrophoresis. Her family history included a consanguineous Lebanese background, a male sibling with similar infections and a female sibling deceased at age 6 years due to leukaemia. At diagnosis her baseline immunoglobulins included IgG 3.2 g/L (6.6–15.6), IgA < 0.1 g/L (0.75–3.8) and IgM < 0.25 g/L (0.4–3.1). Immunophenotyping demonstrated CD4 71% (37–63%), CD8 13% (10–39%) and total B cells 4% (5–24%). Within the B cell compartment memory B cell 7.9% (> 11% of B cells), switched memory B cells 0.08% (> 0.4% lymphocytes) and CD21 low B cells 54%.

Whole genome sequencing revealed a pathogenic homozygous variant in *ADA2*, c.1397_1403del p.(Lys466Thrfs*2) [8]. Consistent with a pathogenic variant, serum ADA2 levels were significantly reduced in the patient, 2.9 mU/g protein (> 24.9) [9].

The patient additionally developed intermittent symptoms of immune dysregulation often preceded by infection. Key features included alopecia, small joint inflammatory arthropathy, sicca symptoms, Raynaud’s phenomenon, hepatitis and trilineage cytopenia. Autoimmune serology including anti-nuclear antibodies and extractable nuclear antigen antibodies were negative. The patient was investigated with CT scanning which demonstrated biopsy-proven multi-system granulomatous inflammation affecting the lung, lymph nodes, liver, and spleen. Histology did not demonstrate cellular atypia or angioinvasion with typical epithelioid histiocytes, small lymphocytes and fibrosis. There was no clinical or radiological evidence of a medium vessel vasculitis or autoimmunity. Imaging included CT angiography and Positron emission tomography (PET).

After 6 years of Ig replacement monotherapy with successful control of sinopulmonary bacterial infections, the patient presented in March 2021 with more prominent features of immune dysregulation including small joint arthropathy and alopecia without fevers. Screening for potential causes detected EBV viraemia 1103 copies/mL (reference range: <50). She was treated with a short course of oral prednisolone and hydroxychloroquine for these symptoms. EBV had not been monitored prior to this presentation and remained elevated (255–1728 copies/mL) over the next 2 months. During this period the patient’s symptoms resolved, and she subsequently self-ceased immune suppression and was lost to follow up.

**Fig. 1:**
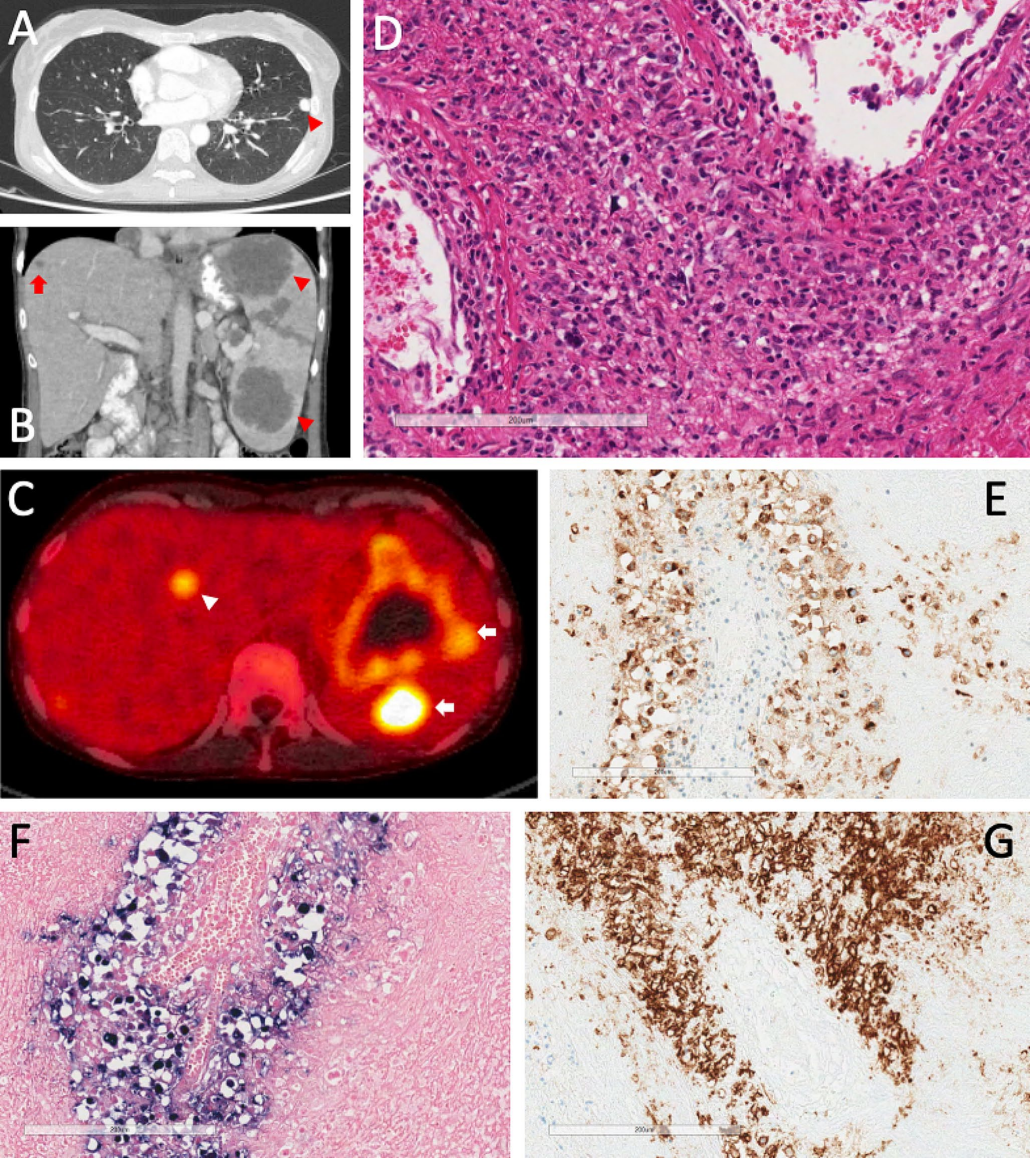
(**a**) CT scan through chest showing pulmonary granulomatous lesion (arrow head). (**b**) CT through abdomen showing lesions affecting the spleen (arrow heads) and liver (arrow). (**c**) PET scan though abdomen showing avid FDG lesions in the spleen (arrows) and liver (arrow head) in both organs. (**d**) Splenic biopsy (haematoxylin/eosin) showing large atypical lymphoid cells with an angiocentric growth. (**e**) Splenic biopsy CD30 stain highlighting the neoplastic B cells. (**f**) Splenic biopsy EBER stain. (**g**) Splenic biopsy CD20 stain showing neoplastic B cells with an angiocentric arrangement

**Fig. 2:**
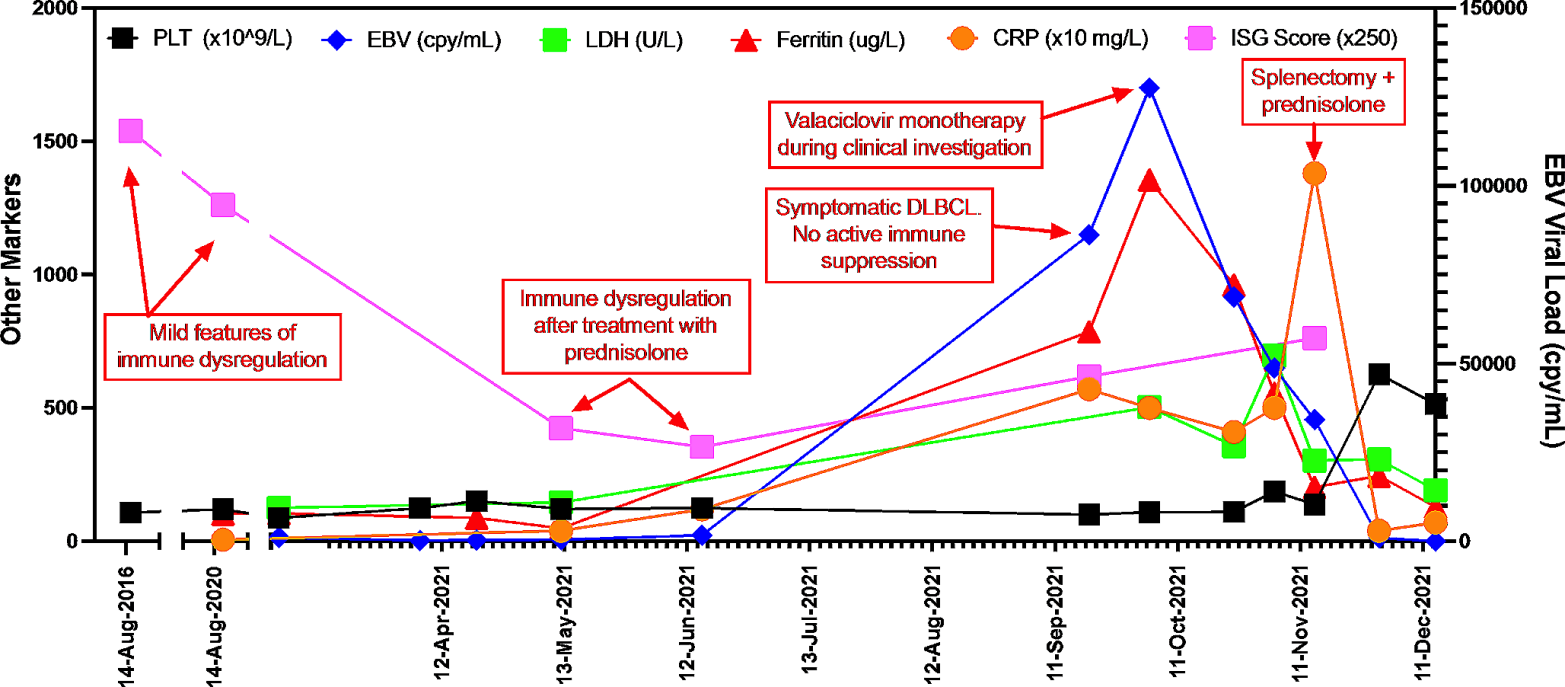
Change in interferon score, EBV viral load and other clinical parameters during clinical presentation plotted against symptoms and key clinical events. Mild immune dysregulation consisted of cytopenias, raynauds and alopecia

She represented in September 2021 with a reported 4-month history of recurrent fevers, 11 kg weight loss and progressive lymphadenopathy. Tri-lineage cytopenia was recognised with reductions in haemoglobin (94 g/L, range: 115–160), neutrophils (1.6 × 10^9^/L, range: 2–8), lymphocytes (0.3 × 10^9^/L, range: 1–4) and platelets (109 × 10^9^/L, range: 150–400). Persistent EBV viraemia was identified with a peak viral load of 127,500 copies/mL. CMV was not detected. Other significant pathology included elevated levels of CRP (50 mg/L; reference range: <5), ESR (36 mm/hr; reference range: <25), LDH (370 U/L; reference range: <250), soluble CD25 (15,096 pg/mL; reference range: <2678) and ferritin (1355 ug/L; reference range: <150). No evidence of a B cell lymphoproliferative disorder was identified by flow cytometric analysis of peripheral blood. The duration of symptoms was not consistent with previous episodes of immune dysregulation. During the diagnostic phase the patient was treated with oral valaciclovir 1 g twice daily initially without other immunomodulatory therapy and a progressive reduction in EBV viral load to < 150 copies/mL was noted. CT imaging demonstrated a significant progression of previously identified granulomatous lesions and development of new lesions affecting the lung, liver and spleen (Fig. 1a and b). PET CT demonstrated multiple hypermetabolic lesions in the liver and spleen with fluorodeoxyglucose (FDG)-avid lymph nodes above and below the diaphragm (Fig. 1c). Core biopsy of the liver demonstrated necrotising granulomatous inflammation with a small rim of atypical lymphoid cells. Bone marrow biopsy demonstrated mildly hypercellular marrow with myeloid and megakaryocytic hyperplasia and reactive lymphoid nodules in keeping with reactive marrow. There was no evidence of haemophagocytosis or lymphoma involvement in the bone marrow sample. Sequential assessment of a type I Interferon gene signature (IFN-stimulated genes; ISG) was performed prior to the development of lymphoma. The ISG score, EBV viral load and timing of key clinical events are presented in Fig. 2. The ISG score represents the median fold change in mRNA expression of six type I ISGs (*IFI27, IFI44L, ISG15, IFIT1, RSAD2, SIGLEC1*) relative to healthy controls as described by Rice et al [10].

Two further spleen biopsies were performed and were suggestive but not diagnostic for EBV related DLBCL. A splenectomy was performed which demonstrated the presence of angiocentric and angiodestructive lymphoproliferative processes with neoplastic cells infiltrating arteries that were intermediate and large pleomorphic cells with vesicular chromatin and prominent nucleoli (Fig. 1d). These large neoplastic cells were EBV positive (Fig. 1f), and expressed CD20 (Fig. 1g), and variable levels of CD30 (Fig. 1e). The morphology and immunophenotype was consistent with EBV positive DLBCL.

The patient was treated with six cycles of R-CHOP21 chemoimmunotherapy (rituximab 375mg/m^2^ IV day 1, cyclophosphamide 750mg/m^2^ IV day1, doxorubicin 50mg/m^2^ IV day 1, vincristine 1.4mg/m^2^ IV day 1, prednisolone 100 mg orally day 1 to day 5 given every 3 weeks) with an additional two doses of rituximab (given 21 days apart) achieving a complete metabolic remission at the end of treatment as assessed by FDG PET. EBV viral load has remained undetected since commencing treatment.

## Discussion

DADA2 is a primary immunodeficiency that can present similarly to CVID without classical features of vasculopathy [1]. While DADA2 is typically considered a humoral immunodeficiency, evidence of a combined phenotype has been identified with increased T cell senescence, impaired survival and aberrant granzyme production by CD8 + T cells [8]. Patients are therefore at risk of opportunistic infections including chronic EBV viraemia and subsequently developing haematological malignancy [1].

EBV related haematological neoplasms including DLBCL can occur in the setting of chronic EBV infection due to impaired T cell surveillance. Lymphomas with similar a phenotype have been identified in other inborn errors of immunity including Wiskott Aldrich Syndrome and X-linked lymphoproliferative disease 1 and DOCK8 deficiency [5, 11, 12].

The prognosis of EBV positive DLBCL in the absence of immunodeficiency is significantly improved when identified at early/premalignant stages, which are responsive to type I IFN therapy [5]. However, due to the IFN gene signature in patients with DADA2, the role of IFN therapy in these circumstances is uncertain, and potentially deleterious [8, 13–15]. Our case demonstrated a positive IFN signature (Fig. 2, Supplementary Figs. 1–6) with an increased ISG score initially without overt evidence of immune dysregulation. Interestingly, clinical symptoms of DLBCL developed after a short course of corticosteroid-induced suppression of the ISG signature following clinical immune dysregulation. It is unclear if the reduction in production of endogenous type I IFN altered the clinical trajectory. Furthermore, given the presence of malignancy and EBV viraemia, the applicability of the detected ISG signature in this case to other patients with DADA2 remains to be completely determined.

In our patient there were initial concerns for hemophagocytic lymphohistiocytosis given the recognised role of EBV in precipitating similar reactions in other inborn errors of immunity. Valaciclovir is an inhibitor of EBV replication and has been suggested to be an adjunct therapy in the treatment of hemophagocytic lymphohistiocytosis and has been shown to inhibit viral shedding [16–18]. In our patient, a continuous reduction in quantitated levels of EBV without the addition of other immunomodulators was noted, with elimination of viraemia over 8 weeks. It is unclear if the treatment of this patient with valaciclovir was responsible for this improvement. However, this clearly did not prevent the onset of lymphoma. There are cases of B cell depletion therapy to prevent overt immune dysregulation, and there may have been a window of opportunity in this case to administer Rituximab early in her presentation to prevent progression to a high-grade lymphoma [17].

## Conclusions

In patients at risk of impaired EBV surveillance, including those with DADA2, consideration should be given to monitoring for the development of EBV viraemia and commencing preventative EBV specific immunotherapy to prevent the development of haematological malignancy. The role of Valaciclovir in preventing EBV replication requires further assessment.

### Electronic Supplementary Material

Below is the link to the electronic supplementary material.

Supplementary Material 1

## Data Availability

No datasets were generated or analysed during the current study.
