# Supplementary material for: Development of EBV Related Diffuse Large B-cell Lymphoma in Deficiency of Adenosine Deaminase 2 with Uncontrolled EBV Infection
Source: J Clin Immunol. 2024 May 17;44(5):118. doi: 10.1007/s10875-024-01712-x (PMC11101521; doi:10.1007/s10875-024-01712-x)
Supplement: Supplementary file 1 — Supplementary Material 1 [file 10875_2024_1712_MOESM1_ESM.docx]

**Supplementary Appendix**


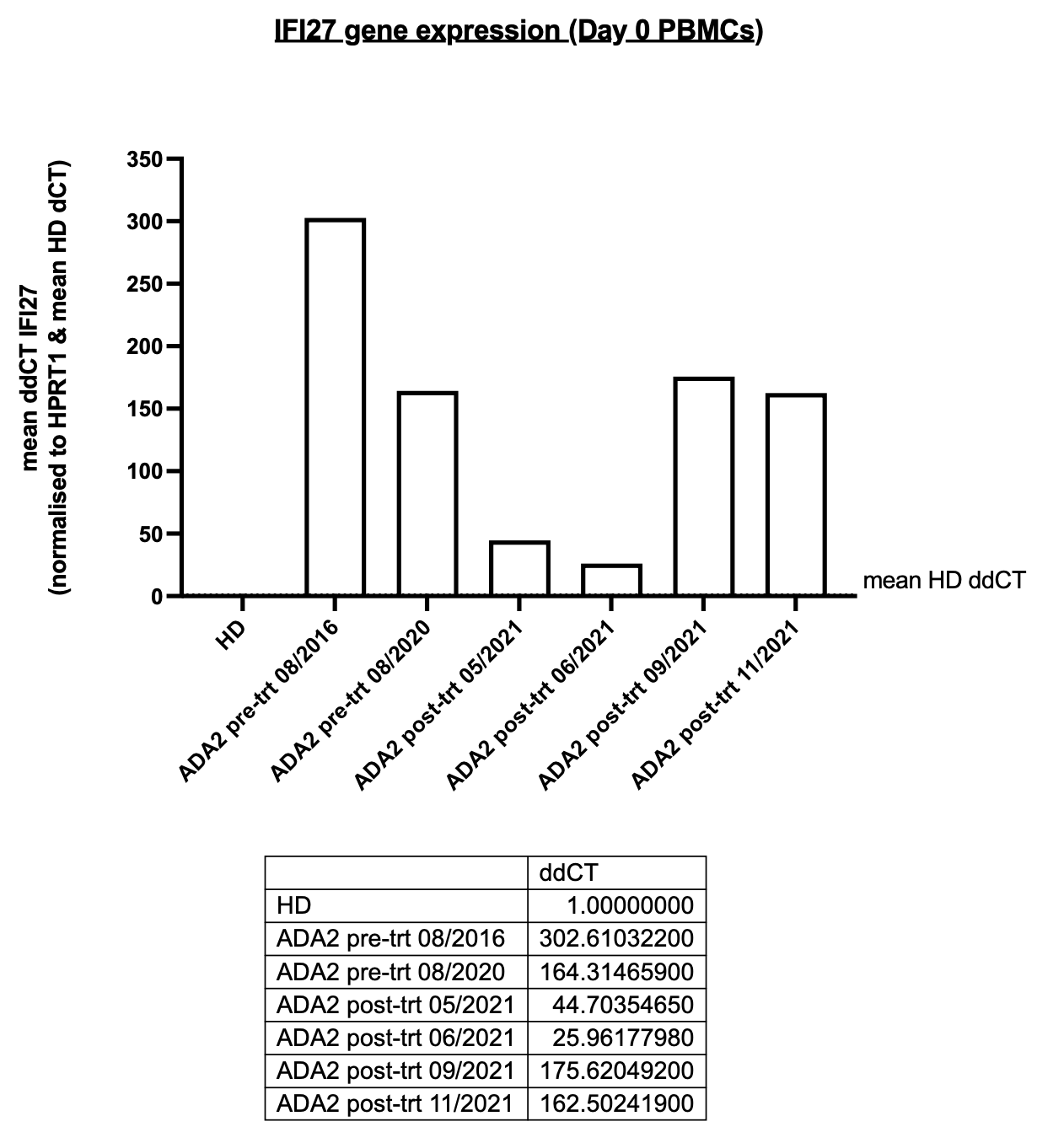


Supplementary Figure 1: IFI27 gene expression at clinical time points


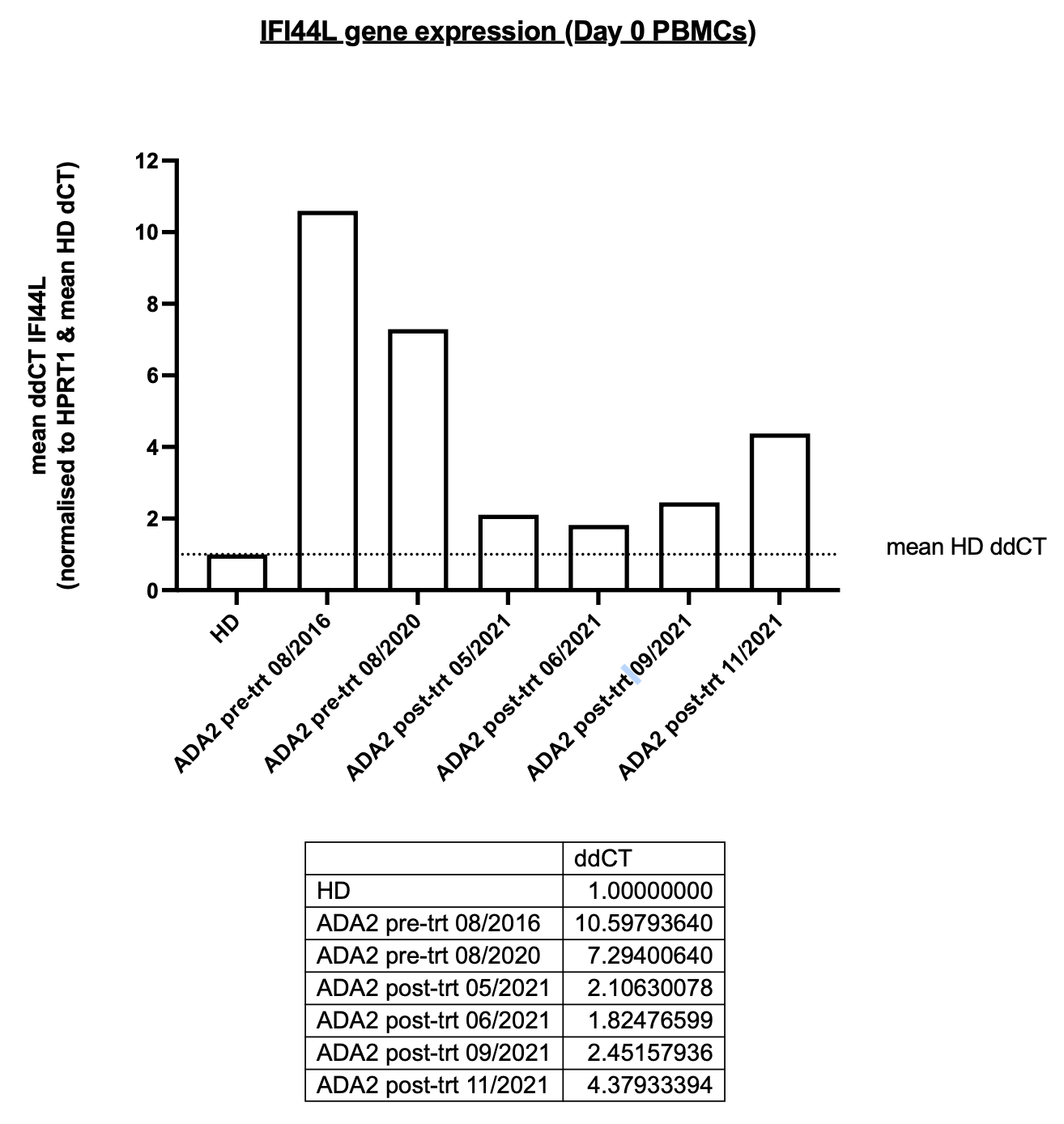


Supplementary Figure 2: IFI44L gene expression at clinical time points


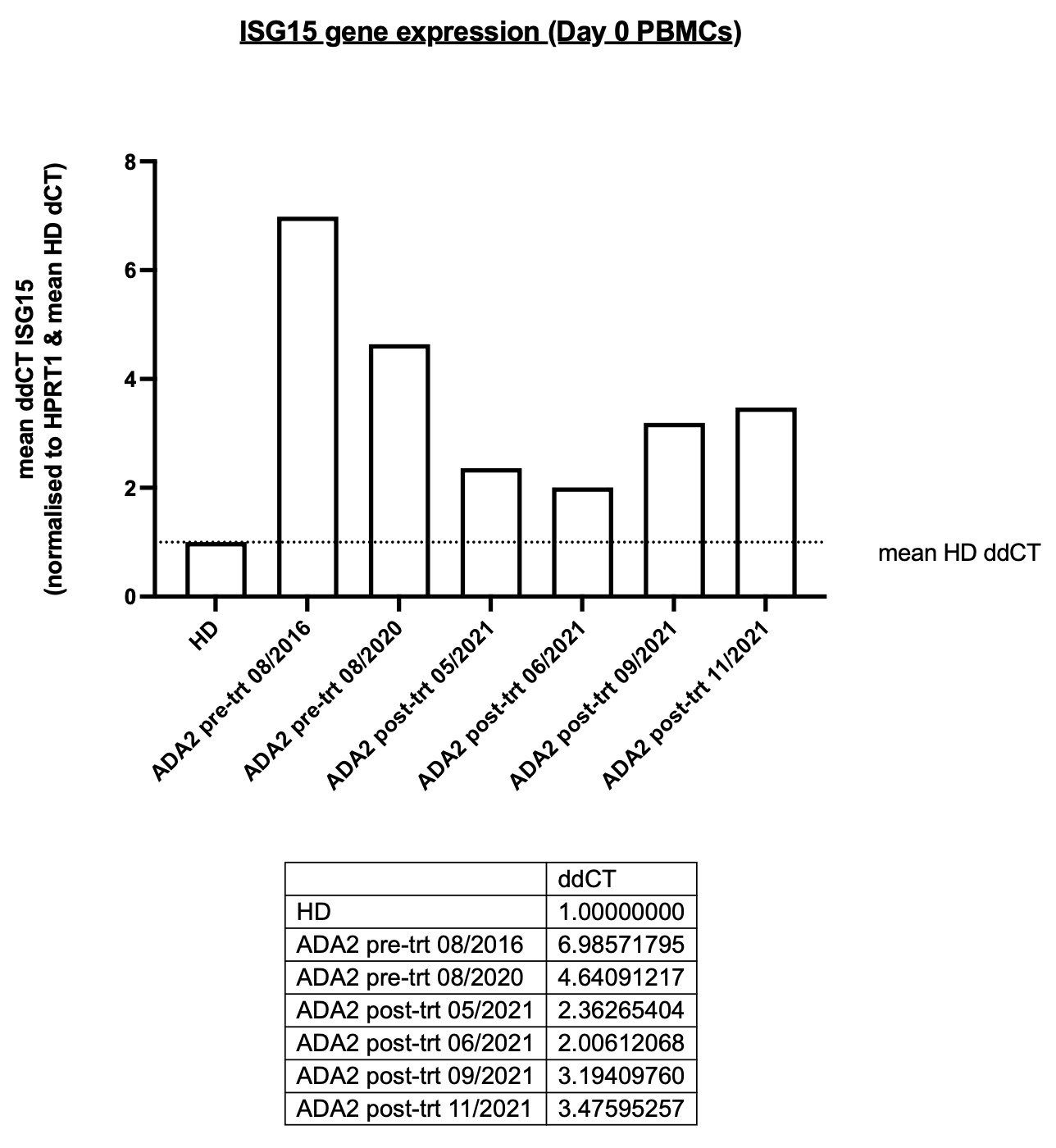


Supplementary Figure 3: ISG15 gene expression at clinical time points


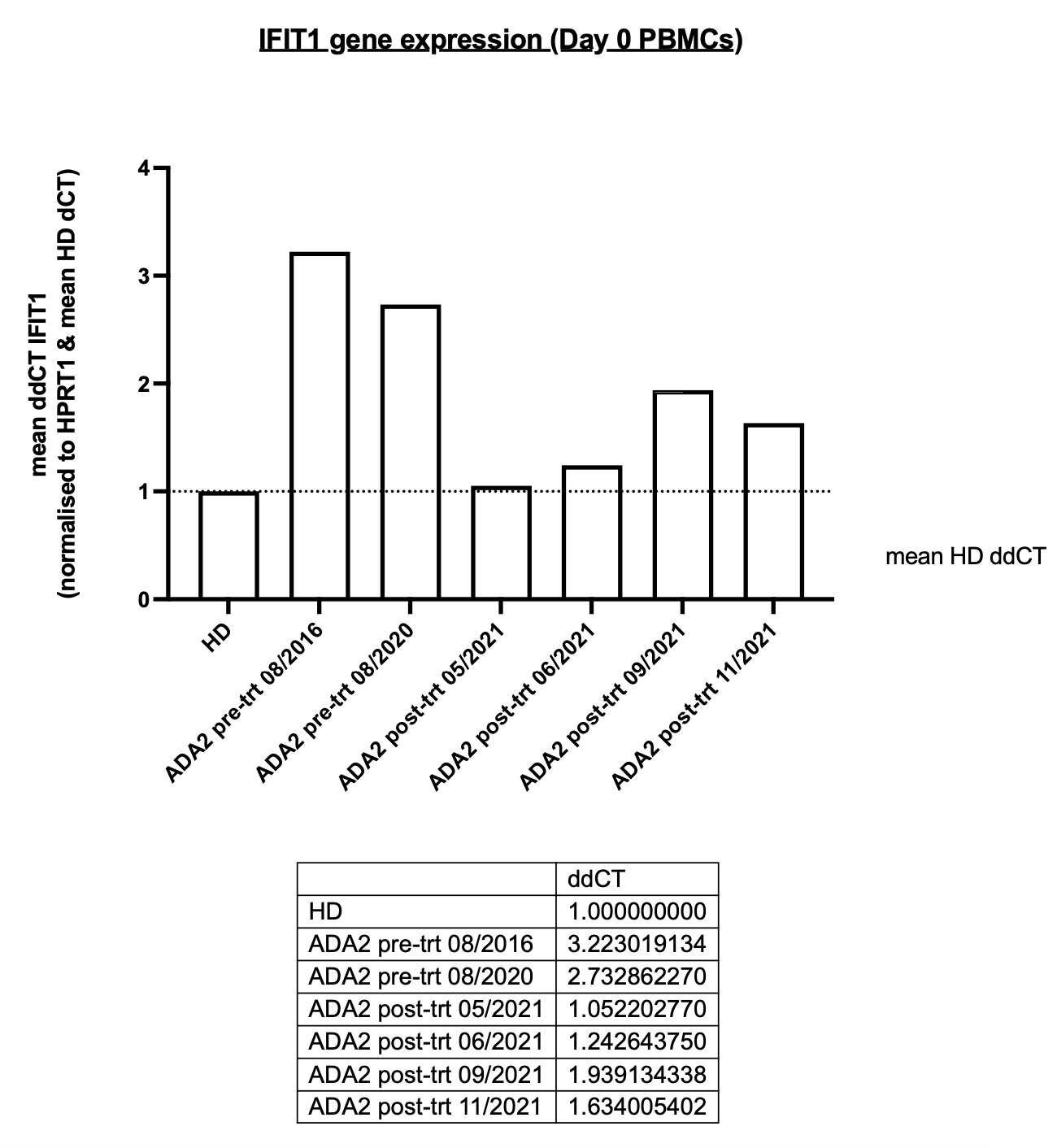


Supplementary Figure 4: IFIT1 gene expression at clinical time points


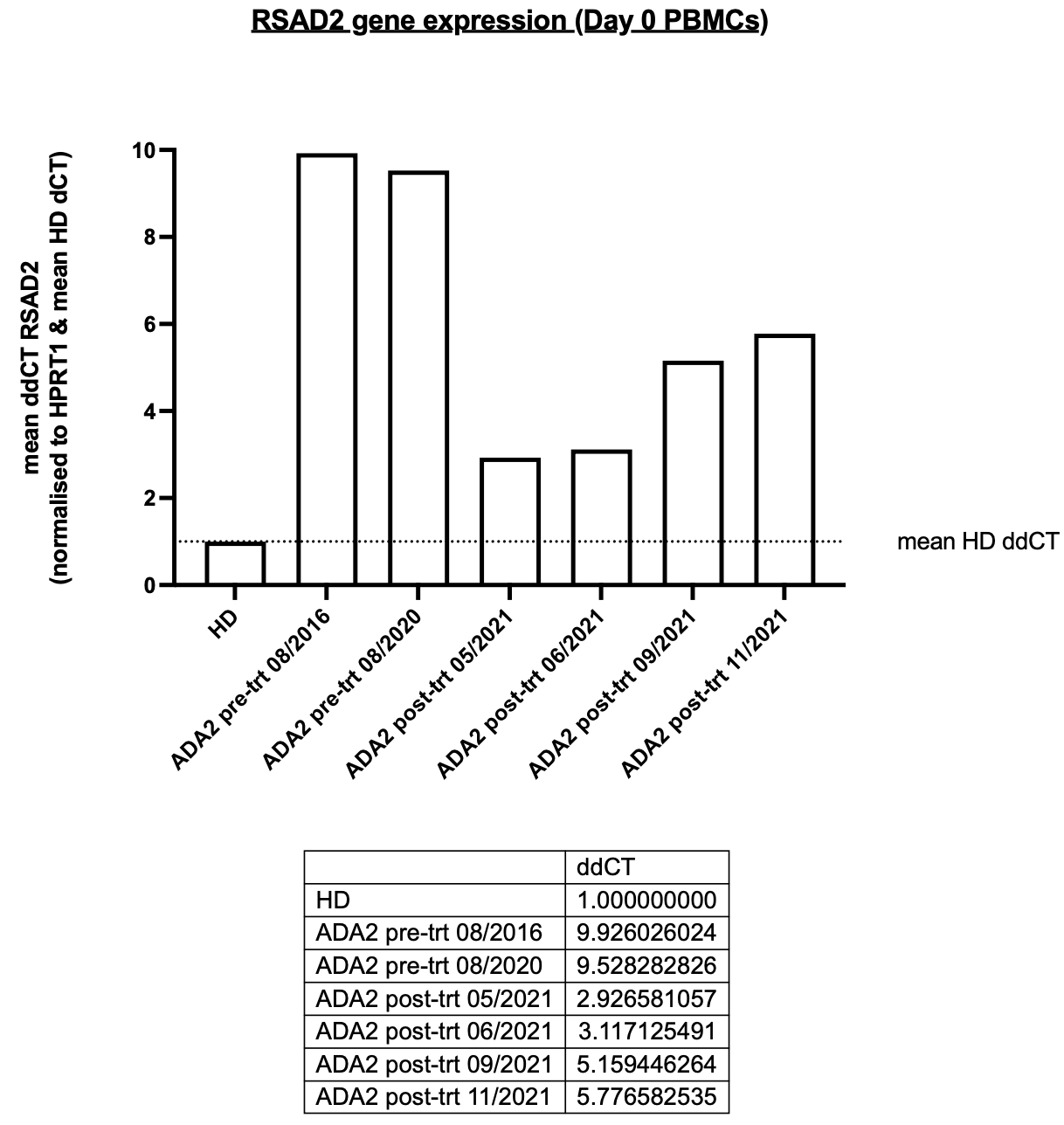


Supplementary Figure 5: RSAD2 gene expression at clinical time points


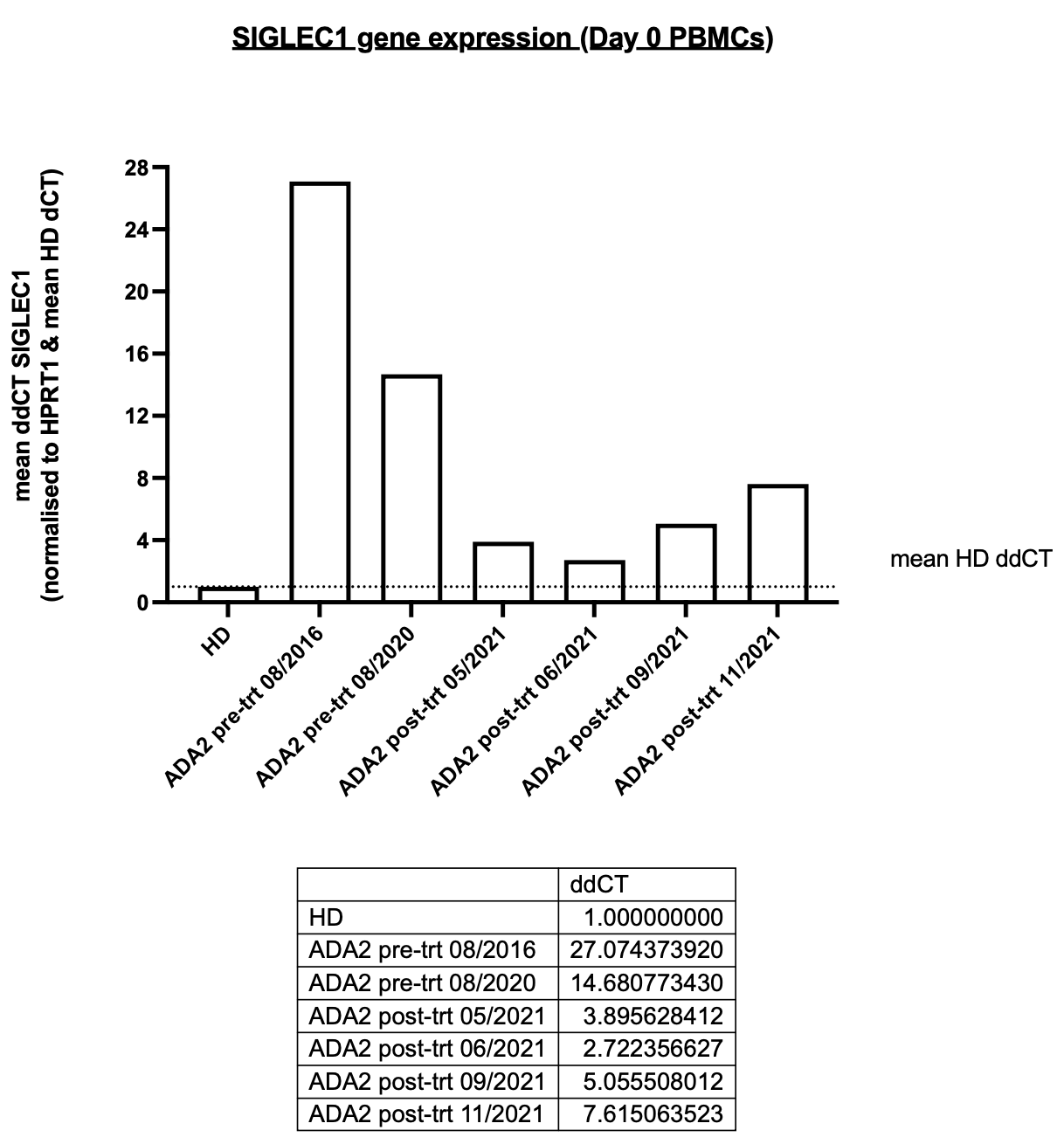


Supplementary Figure 6: SIGLEC1 gene expression at clinical time points
